# Reliability of the pre-operative imaging to assess neck nodal involvement in oral cancer patients, a single-center study

**DOI:** 10.4317/medoral.25228

**Published:** 2022-02-20

**Authors:** Antti L Pakkanen, Emilia Marttila, Satu Apajalahti, Johanna Snäll, Tommy Wilkman

**Affiliations:** 1 MD, DDS. Department of Oral and Maxillofacial Diseases, Helsinki University Hospital and University of Helsinki, Helsinki, Finland; 2 MD, DDS, PhD. Department of Oral and Maxillofacial Diseases, Helsinki University Hospital and University of Helsinki, Helsinki, Finland; 3 DDS, PhD. HUS Medical Imaging Center, Department of Radiology, Helsinki University Hospital, Helsinki, Finland

## Abstract

**Background:**

Primary sites for the metastasis of oral cancer are the cervical lymph nodes. Although there has been considerable technical advancement in the radiological imaging, capability to recognize all metastatic lymph nodes pre-operatively has remained as a challenge. Thus elective neck dissection (END) has remained as reliable practice to treat cervical lymph nodes. This study evaluated the accuracy of pre-operative imaging in pre-operative diagnostics of cervical lymph node status using computed tomography or magnetic resonance imaging in patients with oral squamous cell carcinoma (OSCC). We have also considered the reasons for the difficulties to recognise metastatic nodes in cervical area.

**Material and Methods:**

Patient charts of patients who had had elective neck dissection as a treatment for primary OSCC in the Department of Oral and Maxillofacial Surgery, Helsinki University Hospital between 2016 and 2017 were assessed retrospectively. The outcome variable was post-operatively histologically confirmed lymph node metastasis in the neck area. The primary predictor variable was radiologically confirmed metastasis in the neck area. The explanatory variables were age, sex, pT-class, imaging modality, delay and location of the tumour. Descriptive statistics, sensitivity, specificity and Youden-J index were computed.

**Results:**

Eighty-three patients were included in the study. The sensitivity to detect pathological lymph nodes was 44.8%, and the specificity for the examination was 87.0%. 19.3% of cN0 patients had metastasis in the cervical nodes, whereas of the cN+ patients 8.4% were actually pN0. Patients having cN0, the largest neck metastasis was over 10 mm in 12.5%, whereas cN1-3 patients the corresponding rate was 45.5%. The computational threshold to diagnose a metastatic node was 7 mm.

**Conclusions:**

Especially small metastases are difficult to diagnose. Limitations of radiological diagnostics must be considered when treating OSCC.

** Key words:**Oral cancer, Metastasis, CT, MRI, Neck dissection.

## Introduction

Cervical lymph nodes are the primary site of metastasis in oral carcinoma. Malignant involvement of lymph nodes is the most important independent prognostic factor in oral carcinoma and decreases the 5-year survival significantly (1,2). Previously, the only reliable way to detect cervical lymph node metastases in oral carcinoma was to perform an elective neck dissection (END) (3,4). Other practices have been to reserve neck dissection for a salvage procedure in case of later metastasis. However, patients with salvage neck dissection often have an increased number of positive nodes and extracapsular spread, and therefore, require a more radical neck dissection, resulting in increased morbidity (5,6).

The current accepted practice is to perform an END when the risk of nodal metastasis is estimated to exceed 20% (7). A recent meta-analysis considered END to be superior to observation in patients with early stage cT1/T2N0 tongue cancer (8). However, even if END improved disease-specific survival rate, it remained non-significant for disease-free survival and cervical nodal recurrence. It has also been shown that approximately 70-80% of cN0 oral carcinoma patients receive no benefit from END (5,9,10), and sentinel lymph node biopsy is recommended instead (11). This emphasizes the importance of predicting post-operative staging by pre-operative means as accurately as possible. Particularly important is the reliability of pre-operative cervical lymph node staging.

Contrast-enhanced computed tomography (CT), magnetic resonance imaging (MRI) and ultrasound are standard tools employed in many centres to detect local metastatic lymph nodes. Imaging has been shown to be superior to clinical palpation of the neck area (12). Despite this, clinically and radiologically undetecTable occult metastases are present in up to 20-46% of cN0 oral carcinomas (2,13-15). No consensus has been reached regarding superiority between the different imaging techniques (16,17). According to previous studies, they offer similar diagnostic accuracy in detecting nodal metastases. Pre-operative radiological staging plays a significant role in the choice of treatment offered to patients.

The aim of this retrospective study was to analyse the accuracy of pre-operative imaging in pre-operative diagnostics of cervical lymph node status using CT or MRI in patients with oral squamous cell carcinoma (OSCC). Our hypothesis was that radiological lymph node status predicts the result of histopathological analysis of the neck dissection sample.

## Material and Methods

- Study design

We designed and implemented a cross-sectional retrospective study in which we assessed patient charts of all patients who had had END as a treatment for primary OSCC in the Department of Oral and Maxillofacial Surgery, Helsinki University Hospital between 2016 and 2017.

We included patients with clinically and histopathologically confirmed primary OSCC. Exclusion criteria were prior radiotherapy or chemotherapy, unknown primary tumour or earlier malignancies of the head and neck area.

- Study variables

The outcome variable was post-operatively histologically confirmed lymph node metastasis in the neck area. The primary predictor variable was radiologically confirmed metastasis in the neck area.

In addition, a threshold value for a detected positive lymph node was calculated. The explanatory variables were age, sex, pT-class, imaging modality (CT or MRI or both), delay (time from imaging to surgery) and location of the tumour (according to ICD-10 classification).

- Tumour staging

Diagnosis and tumour staging of all patients were confirmed by the multi-disciplinary head and neck tumour board of Helsinki University Hospital. At least two head and neck radiologists analysed each pre-operative radiological image and the pre-operative staging and treatment plan was decided according to consensus of the multi-disciplinary meeting (Finnish National Guidelines for Treatment of OSCC and TNM Classification of Malignant Tumors 7th ed UICC). The final histopathological diagnosis and staging were set by the multi-disciplinary head and neck tumour team.

- Imaging

MRI was performed using either a 1.5 T unit (Magnetom Vision; Siemens, Erlargen, Germany) or a 3 T unit (Magnetom Vision; Siemens, Erlangen, Germany or Philips Medical Systems). CT was performed using General Electric BrightSpeed or LightSpeed. Both MRI and CT were performed in contrast-enhanced mode. Slice thickness of the reformats ranged from 1.0 mm to 3.0 mm.

- Radiological lymph node status criteria

The lymph node status was based on a radiological statement achieved by pre-operative imaging using CT or MRI or both. The two major imaging criteria used were nodal size and the presence of central nodal necrosis or nodal heterogeneity. We used the minimum axial diameter of the lymph node, with normal nodes not exceeding 11 mm in the jugulodigastric region and 10 mm elsewhere in the neck and head (18-20). In addition, a retropharyngeal node was considered normal if it did not exceed 8 mm in maximum diameter or 5 mm in short-axis diameter (21). Size criteria for metastatic lymph nodes are presented in Table 1.

**Table 1:**
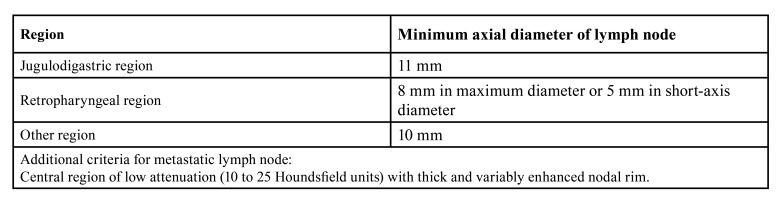
Criteria for metastatic lymph node according to the region of the neck.

On contrast-enhanced imaging, necrotic nodes have a central region of low attenuation (10 to 25 HU) with a thick and variably enhanced nodal rim and usually high signal intensity on T2-weighted images corresponding to necrosis.

- Statistical analysis

Statistical analysis was conducted with IBM SPSS 26. Two-tailed t-test was used to compare descriptive statistics of the groups pN0 and pN1-3. Youden-J index was calculated for patients having metastasis in the neck area. The cut-off value for the size of the detected metastasis was estimated. We estimated the sample size of statistical significance for the observed system with parameters for lowest accepTable sensitivity of 45 % and specificity of 85%, confidence interval of 5% and with 35% of incidence of the metastasis (22).

## Results

A total of 102 patients treated with END for primary oral cancer between 2016 and 2017 were identified from the database of the Department of Oral and Maxillofacial Diseases. Ten patients were excluded for previous surgery of the neck region, 6 patients for malignancy other than oral carcinoma, two patients for unknown primary tumour and one patient due to indistinct radiological statement. Thus, a total of 83 patients were included in the final analyses.

Descriptive statistics of the patients are presented in Table 2. The most common tumour size was pT4. None of the included patients had pT3 tumour. The median ages of patients with pN0 (no cervical metastasis found in histopathological analysis) and pN1-3 (metastasis found in the histopathological analysis) were 68.9 and 70.0 years, respectively. CT and MRI were used to characterize the tumour and the neck area, with CT being the most frequently used. Two patients examined with both CT and MRI were included in the MRI group for further analyses.

Most of the patients received surgery within 30 days of imaging (median 22 days). Comparing the two groups, pN0 vs. pN1-3, the delay was almost equal. Additionally, a delay of over 30 days did not affect the final lymph node status. Other differences between the examined study variables and final lymph node status remained non-significant.

For the whole population with pT1-4 (CT and MRI scan), the sensitivity to detect a real positive lymph node was 44.8% and the specificity to rule out a negative lymph node was 87.0%. 19.3% of cN0 patients had pN+, whereas of the cN+ patients 8.4% were actually pN0 (Table 3).

Comparing CT and MRI, the sensitivity of the former was 45.5% and of the latter 42.9% when we analysed the whole group of pT1-4. The corresponding specificity values were 86.8% and 87.5% (Table 4).

We also conducted a comparison of smaller tumours (pT1 and pT2) and larger tumours (pT4). The sensitivity for detecting a positive lymph node was 33.3% for smaller tumours and 57.1% for larger tumours. On the other hand, the specificity for detecting a positive lymph node was 93.9% for smaller tumours and 76.2% for larger tumours.

**Figure 1:**
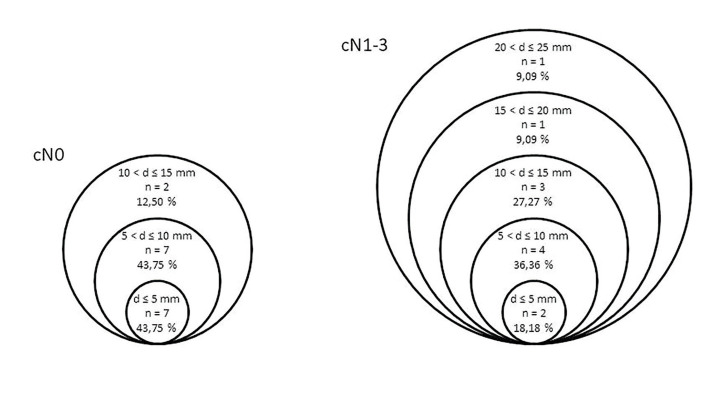
Size of the largest metastatis found post-operatively in histopathological analysis. Patients with pT1-4 examined with CT or MRI scan. Diameter of the largest metastasis is indicated by (d) and number of patients by (n).

Differences between lymph node status and size of the largest histological metastasis are presented in Fig. 1. In 12.5% of patients with cN0, the largest neck metastasis was over 10 mm, whereas in cN1-3 patients the corresponding rate was 45.5%. However, also metastasis of 5 mm or below occurred in this population (10.8%). Using the Youden-J parameter, we estimated that the cut-off value for the radiologist to correctly identify a metastasis in the patient was 7 mm. The sample size for the observed group would have been 1087 patients to gain power to have statistical significance.

**Table 2:**
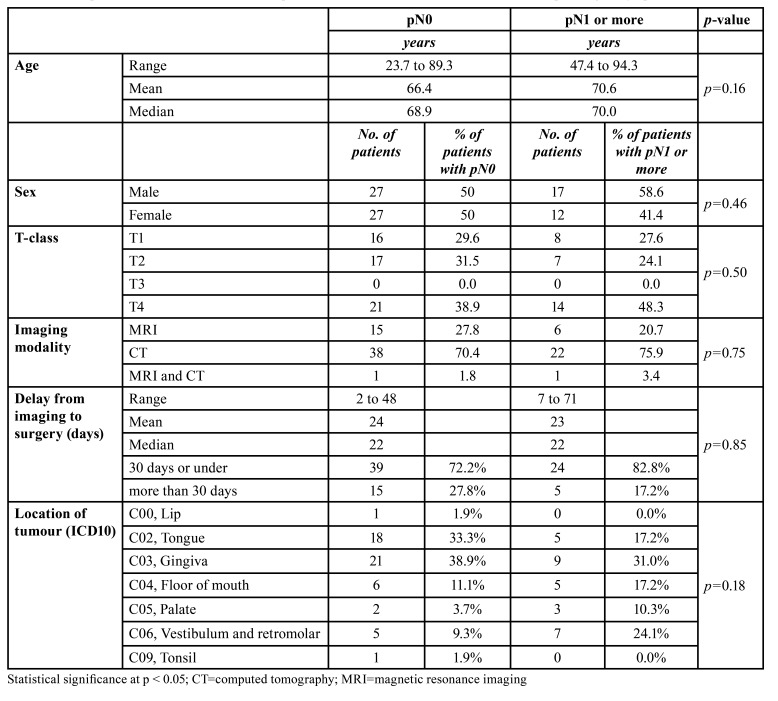
Descriptive statistics of 83 oral carcinoma patients with neck dissection and associations with pathological lymph node status.

**Table 3:**
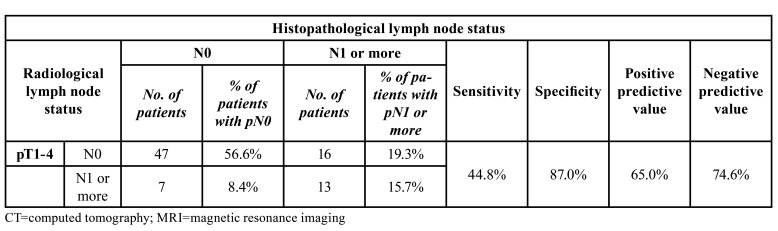
Radiological and histopathological lymph node status in 83 oral carcinoma patients. Sensitivity and specificity for CT and MRI, pT1-4.

**Table 4:**
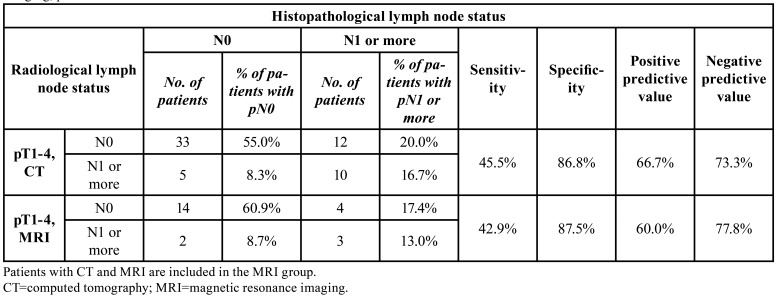
Radiological and histopathological lymph node status in 83 oral carcinoma patients. Sensitivity and specificity for CT or MRI imaging, pT1-4.

## Discussion

**Figure 2:**
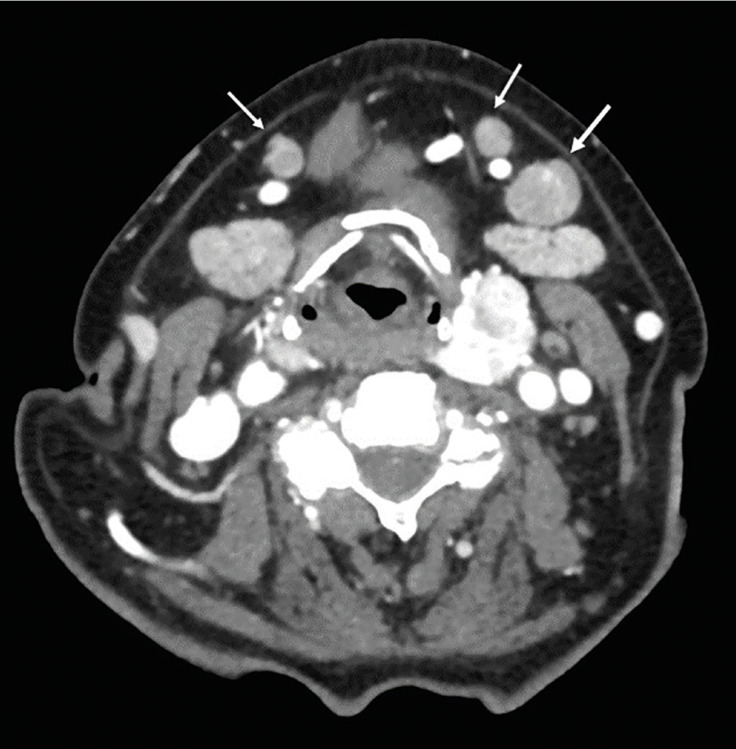
Contrast enhanced computed tomography of a patient with cT3 tongue squamous cell carcinoma on the left side showed a prominent suspicious left submandibular lymph node (large arrow). In addition, smaller rounded lymph nodes were evident bilaterally (small arrows). In spite of radiological findings, metastatic lymph nodes were not detected in postoperative histopathological examination after the elective neck dissection. A prominent thyroid was diagnosed as an incidental finding.

The ability of pre-operative imaging to detect metastatic lymph nodes among patients with OSCC was examined. Our hypothesis was that radiological lymph node status predicts the final cervical lymph node status. The results confirmed the hypothesis only partially. In our population, the sensitivity to diagnose a metastatic lymph node was 44.8% (Fig. 2).

Diagnostics for small metastasis in the neck area remains a challenge. Size criteria for metastatic lymph nodes originate from studies from the 1980s and 1990s (18-20). No noTable changes have occurred in recent years in the diagnostic criteria for metastatic lymph nodes (23). While enormous advances have been made in radiological equipment, occult metastases still occur in 20–40% of patients with OSCC (24). Our study highlights the challenges in clinical decision-making, as a reliable method for assessing cervical lymph node status remains to be developed (6).

Earlier studies have indicated the superiority of imaging over clinical examination and palpation (6). The sensitivity of CT ranges from 47% to 80% and the specificity from 88.9% to 99% (17,25), whereas the sensitivity of MRI ranges from 57% to 93% and the specificity from 82.5% to 99% (16,17,25). PET-CT (Positron Emission Tomography Computed Tomography) has not affected the outcome of imaging significantly (23). Our results highlight the limitations in predicting status of metastatic lymph nodes in the neck area. Up to 44% of cN0 patients have been shown to have occult metastases (23).

In our study, 19.3% of cN0 patients had pN+, whereas of the cN+ patients 8.4% were actually pN0. Most of the nodes that were not detected in pre-operative imaging were smaller than 10 mm. According to our results, the difficulty in assessing metastatic lymph nodes appears to be related to the size of the metastasis. The computational threshold for an undetected metastasis was 7 mm. These findings were consistent even though the CT and MRI scans were analysed by highly experienced head and neck radiologists and the treatment plans were assessed in a multi-disciplinary meeting.

Although there are challenges in pre-operative radiological evaluation and in detection of small metastases, particularly those under 7 mm, pre-operative imaging yields valuable information. Tumour diameter and depth of invasion correlate with histopathological data (26), and imaging is an important component in treatment planning. Skip metastases are rarely seen in neck regions, and thus, one can consider not treating LIV and LV regions in patients without suspicious lymph nodes in pre-operative imaging (24). Additionally, pre-operative imaging helps the surgeon to dissect metastatic lymph nodes involving vital structures in the neck area.

Our study showed higher N1-3 sensitivity in T4 patients than in patients with other tumours. Depth of invasion, micro-metastases and type of growth of the tumour have an effect on the lymph node status found in histological analysis (23). Therefore, it can be assumed that the radiologist is prone to upgrade a suspicious node that would be classified as N0 in a smaller T class. Surgical delay may also lead to differences between radiological staging and final lymph node status. However, in this study, we did not find any significant association between delay of surgery and the final staging.

We examined our patients with up-to-date radiological equipment and used a consistent treatment protocol. Nevertheless, occult metastases were detected. CT and MRI clearly have limitations. Ultrasound scan and PET-CT have been also used to detect metastatic lymph nodes in the head and neck area, but have not proven superior (23,27). Investigations of artificial intelligence in the analysis of radiological data are underway (28).

The main limitation of our study is its retrospective study design. The size of our material also sets limitations on comparison of subgroups with statistical significance, thus the study has to be considered as descriptive. Moreover, we cannot prove that a lymph node detected radiologically is the same one that is diagnosed in histological analysis.

In sum, our study supports the current praxis of treating cervical lymph nodes with END while treating OSCC. Improvements in radiological diagnostics are needed to detect occult metastasis in OSCC. The limitations of radiological diagnostics must be considered when devising a surgical treatment plan.
